# Distributed Solutions for a Reliable Data-Driven Transformation of Healthcare Management and Research

**DOI:** 10.3389/fpubh.2021.710462

**Published:** 2021-07-07

**Authors:** Francesco Sanmarchi, Fabrizio Toscano, Mattia Fattorini, Andrea Bucci, Davide Golinelli

**Affiliations:** ^1^Department of Biomedical and Neuromotor Sciences, University of Bologna, Bologna, Italy; ^2^Department of Internal Medicine, Montefiore Medical Center, New York City, NY, United States; ^3^Department of Preventive Medicine, Azienda USL Toscana Sud Est, Arezzo, Italy; ^4^Department of Economics, G. d'Annunzio University of Chieti-Pescara, Pescara, Italy; ^5^Department of Biomedical and Neuromotor Sciences, University of Bologna, Bologna, Italy

**Keywords:** personalized and precision medicine, blockchain, healthcare management, healthcare research and development, distributed ledger technologies, data, reliability

## Introduction

Modern healthcare management and clinical practice strongly rely on data and scientific evidence. The healthcare sector is a problem-driven, data-intensive domain where the ability to access, edit and trust data emerging from its activities are critical for the operations of the sector as a whole (1). As a consequence, digital technologies, tools, and services became a core component of both Healthcare Management and scientific Research (HMR), as confirmed by the WHO guideline and recommendations on digital interventions for health system strengthening (2). Particularly, HMR is increasingly adopting digital tools that allow data to be shared between geographically distant subjects (3–7). Data interoperability, security, privacy and ease of sharing are core components of high quality HMR. A panoply of solutions is already available to create, collect and share different types of data (6–9) originating from different sources (clinical records, current or *ad hoc* data streams, directly from patients or published studies).

Current data management software in HMR are primarily built on two technological infrastructures: Cloud-Based (CB) or Distributed Ledger Systems (DLTs). Emerging DLTs technologies offer alternative solutions for the management and sharing of data in HMR and can help increase trust in data's integrity and the resulting evidence.

As pointed out by many authors, applications of DLTs in HMR are growing rapidly (1, 10) and they will affect the healthcare domain, specifically patient-centered care (e.g., data ownership and personal health records), and healthcare and research management (e.g., regulatory compliance, providing a decentralized framework for sharing Electronic Health Records (EHR), clinical trials).

The aim of this paper is to shed light on CB and DLT solutions, emphasizing the potential role of innovative digital solutions based on DLTs in creating a data-driven transformation of HMR, and to describe relevant examples and practical uses of DLT-based solutions for patients, healthcare management, and research activities.

## Cloud- and DLT-Based Systems

CB applications are increasingly used for sharing personal data between different subjects in healthcare organizations (11) and in research settings. The benefits of CB tools in HMR have been listed and discussed at length (12, 13). While CB applications are providing new ways of improving HMR processes, they also imply new risks related to the reliability and security of the processed data (14). CB solutions are often managed and run by third parties (i.e., the service providers). As a consequence, control over data is effectively left in the hands of such providers that, being profit oriented, do not necessarily pursue the same interests of patients or HMR professionals and researchers, as it happened with several scandals during the COVID-19 pandemic (14). This increases the probability that personal health data are used for purposes other than those for which they were created (15). Moreover, although data can be kept encrypted in servers, in most systems the user does not have the ability to decide who holds the encryption keys and how these are shared with those having access to the data. Moreover, CB systems pose risks of information leaks, information manipulation, information loss and unauthorized third parties access to data (16).

Alternative technologies for data management and validation in HMR are emerging. One of these is represented by distributed ledger technologies (DLT). Public (Permissionless), consortium (Public permissioned) and private (Permissioned) DLT solutions have been proposed. Public DLT solutions, of which the most famous and widespread is represented by the Bitcoin's Blockchain (17), are open, do not have a “property” or a third party authorization, and are designed not to be controlled by any single entity, but to rely on a system that allows anyone to read or write transactions. This DLT model prevents any form of censorship as no one is in a position to prevent or erase an operation on the ledger once such operation has been approved through the DLT model's consensus mechanism. However, this solution has some disadvantages. Public DLTs can be used as a global database for documents that need to be immutable over time, except for security and privacy updates. They require that all data, or its representation through the so called hashes (i.e., a function that meets the encrypted demands needed to solve for a blockchain computation), are made available to a large number of users. They also require considerable computing power and consequent proportional energy consumption. This implies that current DLT techniques cannot be used in contexts in which the data must be kept confidential and/or the number of users is very limited, such in research studies or in healthcare organizations (18).

On the contrary, consortium or private DLTs rely on closed or private networks. Private DLTs are populated by a series of actors who must rigorously share the same rules. In HMR applications, a consortium or private DLT solution is indicated as a promising alternative (18, 19).

## State of the Art and Perspectives in the Use of DLTs in Healthcare Management and Research

Recently, there has been an effort to develop new applications able to sum up the advantages of the CB and DLTs solutions. The implementation of combined CB/DLTs solutions in HRM opens opportunities in patient-centered healthcare, data analytics, and data transfer. It also helps solve many of the existing challenges in healthcare data management, such as eliminating information silos, increasing efficiency in data transfer, sharing, and protection (20), while guaranteeing the integrity and authenticity of data without the need for a certifying third party.

In the following paragraphs we describe some relevant examples and practical uses of CB/DLT-based solutions in healthcare management and research, focusing on patients, healthcare management and research activities.

### Patients

Plenty of literature suggests that one of the biggest benefits of employing DLT techniques in healthcare, is the enhancement of the so-called patient-centric healthcare. This approach improves the patients' understanding of their condition, increasing concordance and adherence, while improving health outcomes (21–23).

One major issue that could be addressed by DLT solutions is personal data or Personal Health Records (PHR) ownership. A PHR is an electronic record of an individual's health information by which the individual controls access to the information and may have the ability to manage, track, and participate in. A PHR should not be confused with an Electronic Medical Record (EMR). An EMR is held and maintained by a healthcare provider and may contain all the information that once existed in a patient's paper medical record, but in electronic form.

Patient-centric healthcare is made difficult by the current healthcare data infrastructure. EMRs were simply not designed with the patient in mind, and their workflows and interfaces completely exclude the patient. Flipping the current infrastructure around to accommodate patient-centric healthcare would require systems' redesigns. At this end, DLT techniques could allow patients to access their records whenever and to whomever they want (23).

DLTs systems are regarded as superior to cloud systems in terms of privacy and, in some cases, of security. DLTs allow patients to easily take control over their data's permissions, andshare and control data with legitimate users in compliance with terms and conditions set by the data owner (24). In this context, implementing a private DLT infrastructure would be the optimal solution. Private DLTs are populated by a series of actors who must rigorously share the same rules. This can easily comply with GDPR directives since the transactions of the digital records of the stored information can be modified and erased only by the aforementioned actors (25).

Nowadays, patients owned data also means medical devices' originated data. The number of digital medical devices is increasing rapidly and so is the quantity of PHR that those devices are generating. The global Internet of Things (IoT) in healthcare market size is expected to grow from USD 72.5 billion in 2020 to USD 188.2 billion by 2025 (26). An enormous amount of data will be generated in the upcoming years. The immutable data provenance records about medical devices (including software-as-a-medical devices, SaMDs), which can be guaranteed by DLTs, may be helpful for the patients, physicians, and healthcare facilities to procure highly accurate and trustworthy information from the medical devices (24).

These technologies can provide a safe and reliable access to patients' PHR while ensuring and tracking their quality and security. Patients' will finally have complete control over their health data and will be able to share it with entrusted partners and institutions only. Thus, they will be sure that the data will be used according to their will and their needs.

### Healthcare Management

#### DLTs Can Also Improve and Innovate Multiple Aspects of Healthcare Management

During the past years, healthcare organizations have pivoted to virtual care. This trend exposes the need for reliable and agile ways to manage patient consent. Google has recently developed the “Cloud healthcare API,” a tool embedded in their cloud system whose main purpose is to facilitate sharing of PHR in their ecosystem. However, DLTs might simplify patients' consent management. Using DLTs and through several peers belonging to different participating organizations, the consent management is assured and protected. Moreover, the intrinsic immutability and traceability features of DLTs can assist to conduct audits to verify compliance with consent management policies.

Linked to the aspects of patient consent management, data privacy and protection must be considered. In order to properly manage sensitive data, it is important to preserve data privacy. DLTs could ensure data privacy and security by providing only authorized access to medical records, while also guaranteeing accountability, access and control of these data (10). A strong emphasis should also be placed on the ability of DLTs to improve team-based care and continuity of care across institutional borders, and identity management and access control across different health systems. These results could be achieved with a DLTs-based PHR system that can bridge the gap between the patient and institution-specific EHRs (6, 10).

Another major issue for healthcare management is the one related to data fragmentation and interoperability: patients visit many healthcare institutions during their lives and each one of these institutions registers their EHR. This leads to data fragmentation that limits the information available to practitioners and service providers (23). That, in turn, hinders a good quality of care. Plenty of literature suggests that data sharing between care providers improves the accuracy of diagnoses and reduces errors in treatment plans (27–30). Widely implemented DLT solutions could address this issue ensuring a secure and accessible way to store and share sensitive data. It should also be considered how the pandemic forced changes in healthcare delivery, boosting the uptake of telehealth and telemedicine technology (25).

### Research Activities

The third practical use of DLTs concerns research activities. Many authors advocate for the implementation of DLTs systems in the scientific research field, in order to promote scientific transparency (31). For instance, ScienceMatter will offer its triple-blind peer-review process through a publishing platform that uses the Ethereum blockchain. Authors and reviewers will be unknown to one another, but their activities and reviews will be logged for all to see, and all data around a submission will be open, immutable and time stamped (31).

In the same context, Choudhury et al. (18) developed a decentralized framework for consent management and secondary use of research data. Recently, a study on a blockchain-based software solution for clinical trials was conducted at Stanford University (32). Therefore, DLTs' adoption has been posited to have positive connotations in clinical trial management (19) and remote patient monitoring (33, 34).

## Conclusions

In recent years there has been an increase in HMR stakeholders' willingness to share data and embrace data sharing practices. Consolidated and emerging technologies represent a fundamental tool for data management in modern HMR.

DLTs can be useful for patients to truly have control over their health, for healthcare policymakers to increase the quality of organizational processes, and for research funders, editors and publishers to increase the return on investment while also ensuring the reproducibility of research.

In conclusion, harnessing the potential of these digital technologies is essential to transform healthcare management and research, by enhancing data quality, reliability, and trust.

## Author Contributions

All authors listed have made a substantial, direct and intellectual contribution to the work, and approved it for publication.

## Conflict of Interest

The authors declare that the research was conducted in the absence of any commercial or financial relationships that could be construed as a potential conflict of interest.
